# Papular acantholytic dyskeratosis of the perianal region in a young woman^☆^

**DOI:** 10.1016/j.abd.2020.12.017

**Published:** 2022-07-19

**Authors:** Laura Trujillo Ramirez, Camilo Andres Morales Cardona, Juan Carlos Hiromi Lopez Takegami

**Affiliations:** a Fundacion Universitaria Sanitas (Unisanitas), Bogotá, Colombia; b E.S.E. Hospital Universitario Centro Dermatologico Federico Lleras Acosta, Bogotá, Colombia

Dear Editor,

Papular Acantholytic Dyskeratosis (PAD) is an uncommon, chronic and recurrent dermatosis of unknown etiology, considered a possible variant of Hailey-Hailey Disease (HHD).^1^, ^2^, ^3^, ^4^

We present a 25-year-old woman with an 18-month history of intense itch associated with perianal papules; previously diagnosed with condylomas treated with emollients, imiquimod 5% cream, and trichloroacetic acid without improvement. She denied a history of venereal disease or sexual risk behaviors and also reported that her father, paternal grandmother, and uncles, had been diagnosed with HHD (Fig. 1).

**Figure 1:**
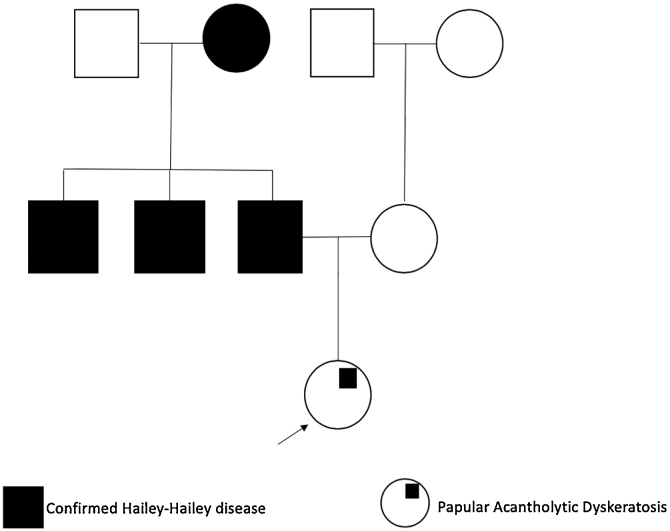
Family pedigree showing patient relatives with HHD confirmed diagnosis.

Physical examination showed multiple grayish-white, keratotic papules in the perianal area (Fig. 2). There were no similar lesions in other body regions, mucous membranes, or nail affectation.

**Figure 2:**
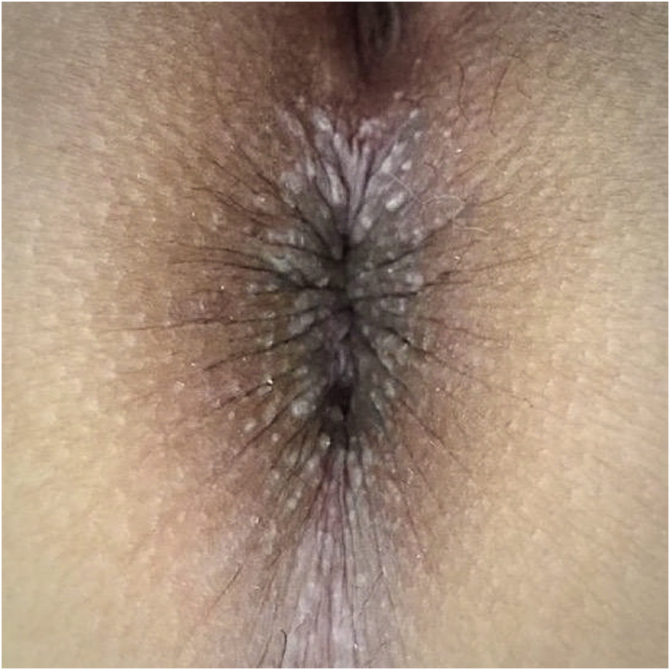
Multiple grayish-white, keratotic papules in the perianal area and perineum.

A 2-mm punch biopsy revealed hyperkeratosis, hypergranulosis, prominent dyskeratosis, acantholysis, and suprabasal clefts (Fig. 3). Correlating her family history and clinicopathological findings, a diagnosis of PAD was made. Topical treatment with tacrolimus 0.1% daily provided itching relief, with clinical persistence of the papules.

**Figure 3:**
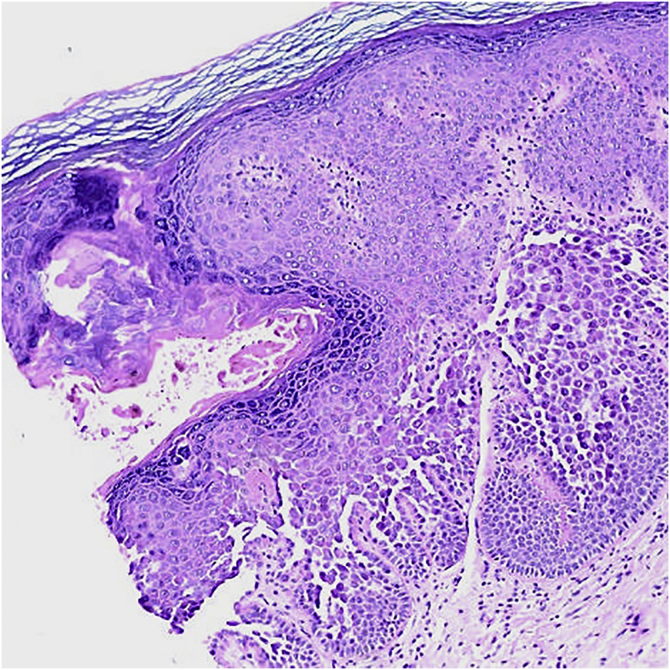
Histopathological findings. Area of dyskeratosis, acantholysis and suprabasal clefts (Hematoxylin & eosin, ×20).

PAD of the genitocrural area is most frequent in young women. Clinical findings are characterized by multiple pruritic grayish, whitish, or erythematous verrucous papules that can be solitary or coalesce in plaques. Papules are usually located on warm moist areas such as the perineum, penis, scrotum, vulva, and perianal or inguinal folds.^1^, ^5^ Most lesions are asymptomatic, but some could be painful or itchy as in this case.^1^

Histology shows hyperkeratosis, focal parakeratosis, acantholytic and dyskeratotic cells in the mid and lower epidermis.^4^ The main histological differential diagnoses are HHD, warty dyskeratoma, and Darier Disease (DD) as they share similar histopathological features included in the spectrum of focal acantholytic dyskeratoses.^1^, ^4^, ^5^ These patterns must be correlated with the patient’s clinical findings and family history in order to ascertain a PAD diagnosis.

HHD is a rare, autosomal dominant inherited genodermatosis, with complete penetrance but variable expressivity.^3^ It is caused by mutation of the ATP2C1 gene (3q21) that codes for the Secretory Pathway Ca^2+^ ATPase type 1 pump (SPCA1).^5^ The non-functional pump gives rise to calcium dysfunction, impairing the correct protein synthesis necessary for desmosome formation, resulting in a keratinocyte adhesion defect.^2^ It is suggested that PAD could be a result of a segmental mosaic mutation of ATP2C1 gene, being a localized atypical variant of HHD.^3^, ^4^, ^5^ Most cases appear to be sporadic, and there are very few reported PAD cases with HHD family history. This disease remains to be fully elucidated and there’s still uncertainty about if it corresponds to an individual entity.^5^

There are various treatment options for PAD including tetracyclines, cryotherapy, systemic and topical retinoids, topical tacrolimus and steroids, CO_2_ laser ablation, or surgical removal.^3^, ^5^ It’s important to consider that the lesions tend to persist for years or can recur after treatment.^3^

Genital dermatoses are frequently focused on as sexually transmitted diseases, especially in young sexually active individuals. PAD diagnosis is challenging, and lesions may be sometimes difficult to distinguish from anogenital warts. This case highlights the importance of knowing about this disease, reducing misdiagnosis, and avoiding unnecessary interventions that could affect the patient’s quality of life.

## Financial support

None declared.

## Authors’ contributions

Laura Trujillo Ramirez: Drafting and editing of the manuscript; conception and planning of the study; intellectual participation in the propaedeutic and/or therapeutic conduct of the studied cases; participation in study design; critical review of the literature; critical review of the manuscript.

Camilo Andres Morales Cardona: Drafting and editing of the manuscript; conception and planning of the study; intellectual participation in the propaedeutic and/or therapeutic conduct of the studied cases; participation in study design; critical review of the literature; critical review of the manuscript.

Juan Carlos Hiromi Lopez Takegami: Drafting and editing of the manuscript; conception and planning of the study; intellectual participation in the propaedeutic and/or therapeutic conduct of the studied cases; participation in study design; critical review of the literature; critical review of the manuscript.

## Conflicts of interest

None declared.
